# Infrared Polariscopy Imaging of Linear Polymeric Patterns with a Focal Plane Array

**DOI:** 10.3390/nano9050732

**Published:** 2019-05-13

**Authors:** Reo Honda, Meguya Ryu, Masayuki Moritake, Armandas Balčytis, Vygantas Mizeikis, Jitraporn Vongsvivut, Mark J. Tobin, Dominique Appadoo, Jing-Liang Li, Soon Hock Ng, Saulius Juodkazis, Junko Morikawa

**Affiliations:** 1 Tokyo Institute of Technology, Meguro-ku, Tokyo 152-8550, Japan; honda.r.aa@m.titech.ac.jp (R.H.); ryu.m.ab@m.titech.ac.jp (M.R.); moritake.m.aa@m.titech.ac.jp (M.M.); 2 Swinburne University of Technology, John Street, Hawthorn, Victoria 3122, Australia; armandas.balcytis@gmail.com (A.B.); soonhockng@swin.edu.au (S.H.N.); 3 Research Institute of Electronics, Shizuoka University, Naka-ku, 3-5-3-1 Johoku, Hamamatsu, Shizuoka 4328561, Japan; mizeikis.vygantas@shizuoka.ac.jp; 4 Infrared Microspectroscopy Beamline, Australian Synchrotron, Clayton, Victoria 3168, Australia; jitrapov@ansto.gov.au (J.V.); tobinm@ansto.gov.au (M.J.T.); 5 THz-Far Infrared Beamline, Australian Synchrotron, Clayton, Victoria 3168, Australia; Dominique.APPADOO@ansto.gov.au; 6 Institute for Frontier Materials, Deakin University, Geelong, Victoria 3220, Australia; jingliang.li@deakin.edu.au; 7 Tokyo Tech World Research Hub Initiative (WRHI), School of Materials and Chemical Technology, Tokyo Institute of Technology, 2-12-1, Ookayama, Meguro-ku, Tokyo 152-8550, Japan; 8 Melbourne Center for Nanofabrication, Australian National Fabrication Facility, Clayton, Victoria 3168, Australia

**Keywords:** focal plane array, thermal source, synchrotron radiation, infrared spectroscopy, hyperspectral imaging, silk

## Abstract

Polariscopy is demonstrated using hyperspectral imaging with a focal plane array (FPA) detector in the infrared (IR) spectral region under illumination by thermal and synchrotron light sources. FPA Fourier-transform IR (FTIR) imaging microspectroscopy is useful for monitoring real time changes at specific absorption bands when combined with a high brightness synchrotron source. In this study, several types of samples with unique structural motifs were selected and used for assessing the capability of polariscopy under this FPA-FTIR imaging technique. It was shown that the time required for polariscopy at IR wavelengths can be substantially reduced by the FPA-FTIR imaging approach. By using natural and laser fabricated polymers with sub-wavelength features, alignment of absorbing molecular dipoles and higher order patterns (laser fabricated structures) were revealed. Spectral polariscopy at the absorption peaks can reveal the orientation of sub-wavelength patterns (even when they are not spatially resolved) or the orientation of the absorbing dipoles.

## 1. Introduction

For the spectral mapping of processes and material modifications in real time, data acquisition with a focal plane array (FPA) becomes a prerequisite in infrared (IR) microspectroscopy. This is particularly important for spectral characterisation of materials using Fourier transform IR (FTIR) microspectroscopic systems at synchrotron facilities where the extracted IR beam possesses the highest brightness [1]. Polariscopy measurements to establish the polarisation state of light in the long IR-THz spectral range is still at an early stage of development, mainly due to the lack of optical elements, filters, and waveplates. A new concept of metasurfaces is expected to be fruitful since it can provide arbitrary amplitude and phase engineering in the plane of the optical element [2]. The complex polarisation of synchrotron radiation is another challenge for maximising all the available luminous power for transmission measurements using polarised light [3]. Fabrication of IR polarisation elements do not require high resolution lithography and is challenging only due to the required deep etching of high-aspect-ratio patterns. The key benefit of polarisation optical elements is in the augmented capabilities of FTIR spectroscopy, where polarisation control allows for a determination of the alignment and orientation of absorbing dipoles.

In this study, we tested the potential of polariscopy with an FPA detector on FTIR microspectroscopic systems for hyperspectral imaging using a thermal IR (Globar^TM^) source and synchrotron radiation [4]. Samples with unique structural motifs were selected for the tests, including brown silk (representative of linear bio-polymer) [5,6,7,8], a laser-polymerised micro-optical element with a polymeric grating motif, and a concentric grating in gold as a reference sample. It was found that imaging parameters (such as the focal spot size and the pixel size) played a critical role in interpreting the FPA-FTIR images obtained from these sub-wavelength features. Based on the results presented here, the FPA-FTIR technique demonstrated its potential as a powerful analytical tool to underpin the research in the design of IR optical elements and to be adopted for polariscopy measurements in IR wavelengths.

## 2. Materials and Methods

**Samples.** An SZ2080 resist (IESL-FORTH, Heraklion, Greece) was used for the polymerisation of q-plates. Standard development protocols [9] were followed for fabrication on Si_3_N_4_ membranes. A sapphire wafer was mechanically thinned to 30 µm by Tecdia Ltd., Tokyo, Japan. Gold coating was done by magnetron sputtering, and Ga-ion milling (IonLiNE, Raith Ltd., Dortmund, Germany) was used to inscribe the pattern. Silk samples were prepared as described earlier [10].

**FPA-FTIR measurement using a thermal IR source:** The offline FPA-FTIR experiment was performed using a Bruker Hyperion 3000 FTIR microscope (Bruker Optik GmbH, Ettlingen, Germany), equipped with a liquid-N_2_ cooled, 64×64 element FPA detector and a matching 15× objective and condenser (NA=0.40), coupled with a Vertex 70 FTIR spectrometer (Bruker Optik GmbH, Ettlingen, Germany) that was equipped with a thermal (Globar^TM^) IR source [4]. FPA-FTIR images were acquired in transmission mode in the 4000–800 cm^−1^ spectral region as a single FTIR image covering a sampling area of 182×182 µm^2^. Each FTIR spectral image comprised a 64×64 array of spectra resulting from each square of the detectors on the 64×64 element FPA array. As a consequence, a single spectrum in each FTIR image represented molecular information acquired from a ca. 2.8×2.8 µm^2^ area on the sample plane. For each image, high-quality FTIR spectral images were collected at an 8 cm^−1^ resolution, with 64 or 128 co-added scans, Blackman-Harris 3-Term apodisation, Power-Spectrum phase correction, and a zero-filling factor of 2 using OPUS 7.2 imaging software (Bruker Optik GmbH, Ettlingen, Germany). Using the same acquisition parameters, background measurements were taken prior to sample spectral images by focusing on a clean surface area of substrate without the structure.

**FPA-FTIR measurement using synchrotron IR source [4]:** Synchrotron-based hyperspectral imaging measurement was performed on the IR Microspectroscopy (IRM) Beamline at the Australian Synchrotron (Victoria, Australia), using a similar Hyperion 3000 microscope and a Vertex 70v FTIR spectrometer system (Bruker Optik GmbH, Ettlingen, Germany). With this setup, the synchrotron FPA-FTIR images were collected with a matching 36× objective and condenser (NA=0.50; Bruker Optik GmbH, Ettlingen, Germany). Due to the restricted focus size of the synchrotron beam, the FPA detector readout was restricted to 32×32 pixels to ensure that spectra were collected only from the most evenly illuminated portion of the detector. Readout rate was 5 kHz (actual integration time used was 0.2146 ms) with a gain of 3. The FPA detector had a long wavelength cut-off of 850 cm^−1^. Using a 36× objective, the detector pixel pitch was estimated to be 1.11 µm × 1.11 µm in the sample focal plane.

**Polarisers.** Holographic ZnSe wire-grid polarisers (Edmund) were used to set polarisation at the IR spectral range of λ = 4000–750 cm^−1^ (2.5–13.3 µm); the extinction of polarisers were $T^{max}/T^{min}≃$ 150, and transmittance ∼50%.

The absorbance or optical density A=−log(T) spectrum is defined by the absorption coefficient α≡4πκ/λ=2ωκ/c [cm^−1^] for the transmitted light intensity $I_{T}=I_{0}e^{-\alpha d}=I_{0}\times 10^{-OD}$, where *d* is the thickness of the sample, transmittance $T=I_{T}/I_{0}$, OD is the optical density, ω is the cyclic frequency of light, and *c* is the speed. Imaging resolution of the FTIR microscopes used were close to the diffraction limit and was specially tested for the synchrotron radiation [4] setup. This was with λ = 8 µm, and NA=0.6. The resolution determined from the imaging of a polymerised 1951 USAF resolution test pattern was 8.1 µm, as defined by 0.61λ/NA [4].

## 3. Results

Three samples with sub-wavelength features, including (i) a circular metal grating, (ii) a laser-polymerised radial structure, and (iii) natural brown silk, were used for FPA-FTIR microspectroscopic imaging measurements.

### 3.1. Circular Sub-Wavelength Grating

**Figure 1:**
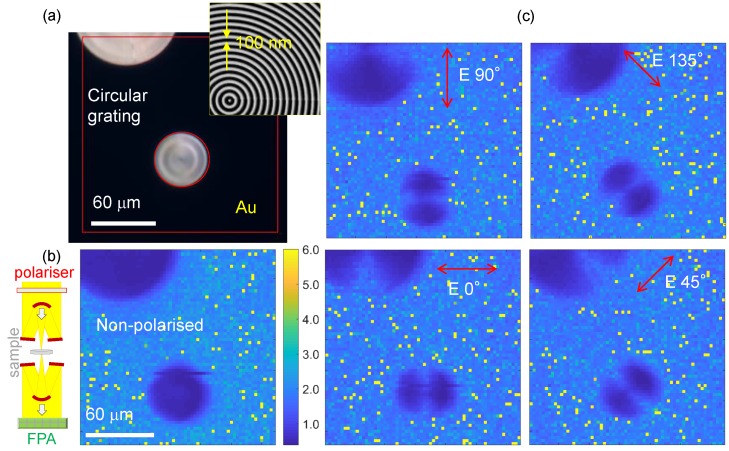
(**a**) Optical image of a circular grating milled through an Au-film on thin sapphire; inset shows SEM image. The grating was milled by Ga^+^-ions in a 100-nm-thick Au film sputtered on 30-µm-thick sapphire; grating: 100-nm-half-pitch. (**b**) Wavenumber 3000 cm^−1^ polarisation dependence of the absorbance A=−log(T), where *T* is transmittance, measured through a circular grating. Inset on the left shows the principle of transmission measurements using a reflective Cassegrainian objective lens with a grid polariser and focal plane array (FPA) imaging. (**c**) Orientation dependence of the absorbance; E-field orientation is marked. The light source was thermal-IR. FPA-FTIR images were taken using 64×64 pixels of the FPA detector. The pixel size was 2.8 µm, the Cassegrainian objective lens magnification was 15×, and numerical aperture NA=0.4 corresponding to the diffraction limit of 0.61λ/NA = 5 µm, a spectral resolution of 8 cm^−1^, and an accumulation of over 128 scans.

**Figure 2:**
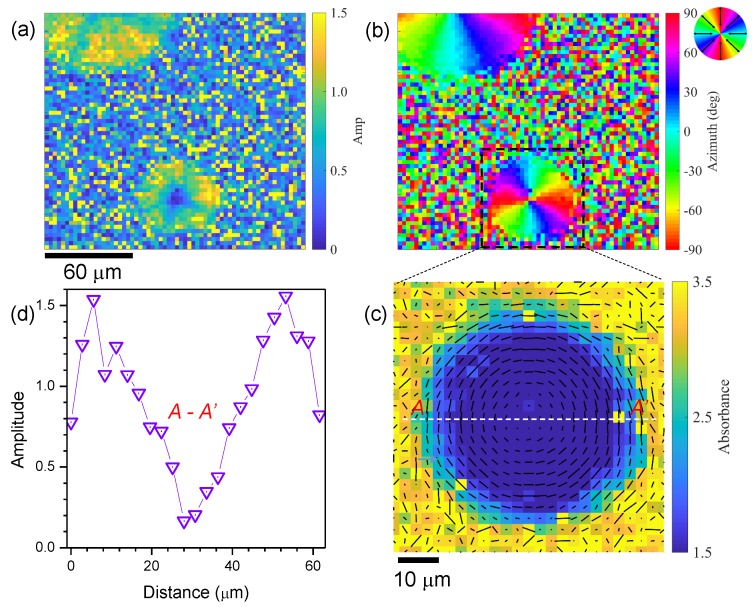
Circular grid reference measured with FPA; thermal-IR source. Orientation amplitude Amp (**a**), orientation azimuth (**b**), absorbance map overlaid with an orientation map (**c**), and central cross section $A-A^{\prime }$ (**d**) all at 3000 cm^−1^.

The first FPA-FTIR experiments were carried out using a thermal IR (Globar^TM^) source. The reference sample was a circle with sub-wavelength grooves with a period of 200 nm and half-pitch spacing of 100 nm. The grating was milled with a focused ion beam (FIB; IonLiNE Raith, Dortmund, Germany) through a 100-nm-thick Au sputtered film, using thin sapphire as a substrate (Figure 1). Transmission *T* at 3000 cm^−1^ (λ = 3.3 µm) was collected at four polarisations of incident thermal radiation. A clear dipolar nature of transmission was observed through the concentric grating; the 100-nm-thick Au film blocked direct transmission. Light with an E-field aligned with the local orientation of the wavevector of the grating k: E‖k had lower absorbance, while a stronger absorbance (lower *T*) was observed in the locations where E⊥k, yielding a dipole pattern. From the absorbance determined at four orientations of linearly polarised illumination, it was possible to determine the orientation of the absorbing species/structures [11]. Using this method, the orientation amplitude and azimuth were determined as summarised in Figure 2. The orientation azimuth angle $\theta^{\prime }$ was measured from the fit taken at four θ angles where the orientation strength is defined by the amplitude of absorbance $A_{max}-A_{min}$ [12]. The strongest orientation amplitude was at the edge of the circular grating where the curvature of the pattern was the lowest. Conversely, in the centre, a less defined orientation amplitude was observed due to the strongest local curvature of the pattern (Figure 2a). The azimuth (Figure 2b) showed the expected circular geometry of the concentric grating as shown in Figure 2c combined with the absorbance plot; the cross section of the absorbance amplitude is shown in Figure 2d.

### 3.2. SZ2080 Polymerised Gratings

**Figure 3:**
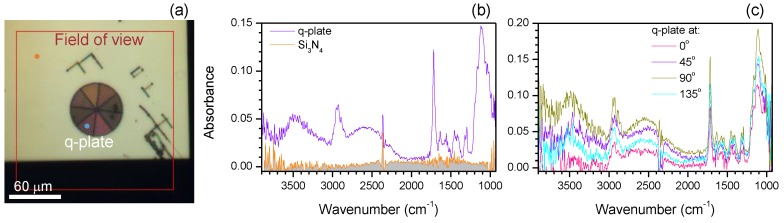
Polarisation dependence of the point spectrum of a q-plate; the light source is thermal-IR. (**a**) Optical image of a q-plate polymerised out of an SZ2080 resist on a Si_3_N_4_ 100-nm membrane. Two coloured markers show the location of the point spectrum measurement shown in (**b**). (**c**) Angular dependence of the point spectrum on the q-plate; θ = 0° is a horizontal orientation.

Micro-optical diffractive elements made of gratings with an azimuthally changing orientation and period of Λ≈400 nm (duty cycle of 0.5) Figure 3a were polymerised by direct laser writing [13]. Such gratings with an azimuthally varying optical axis orientation defined as ψ=qϕ, where *q* is the half-integer, and ϕ is the polar angle in the cylindrical coordinates, called q-plates [14,15]; a usual grating is a q=0 plate, while the concentric grating (Figure 1a) is q=1. Q-plates are used as optical vortex generators [16] applicable to opto-mechanical manipulation of matter [17]. When the propagation phase reaches the λ/2 condition, the most efficient conversion of the incoming spin angular momentum (SAM) σ into the orbital angular momentum (OAM) with a spiralling wavefront is achieved. The azimuthal phase 2qψ defines the topological charge of the optical vortex l=2qσ, where σ=±1 is the spin for the left- or right-hand polarisation, respectively.

Q-plate q=1 was laser-polymerised in a SZ2080 resist with segments of a Λ = 2 µm period grating (Figure 3a). The polymerised bars were approximately 1 µm in width. For operation of the q-plates at IR wavelengths, it is important to have a weak absorbance in the polymer and a height that satisfies π retardation for the propagating phase. Q-plates were polymerised on a 100-nm-thick Si_3_N_4_ membrane. Point spectra from the q-plate and Si_3_N_4_ membrane are shown in Figure 3b. The orientational dependencies at four angles were measured (Figure 3c) to determine the azimuthal distribution of the absorbers [11]. The Si_3_N_4_ nano-membrane showed almost no absorption over the entire spectral window. Spectral features of the SZ2080 at 2923 cm^−1^ (due to C–H stretching in alkanes), 1716 cm^−1^ (due to the C=O stretching of carbonyl), and 2700 cm^−1^ (where there was no specific absorbance in the SZ2080) were selected for the orientation mapping. The Q-plate was polymerised using circularly polarised light at λ=800 nm to avoid polarisation-induced effects in the width of the bars, which became larger along the direction of the polymerisation and depended on the direction of laser writing [18].

**Figure 4:**
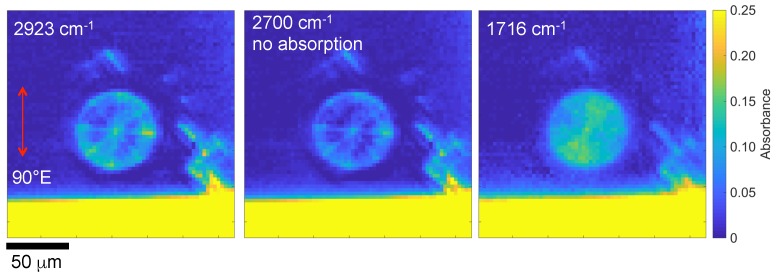
Absorbance images taken using a thermal IR source and the FPA detector at different wavenumbers for the vertically polarised E-field. Note the same scale of absorbance used for comparison. An optical image of the sample is shown in Figure 3a.

**Figure 5:**
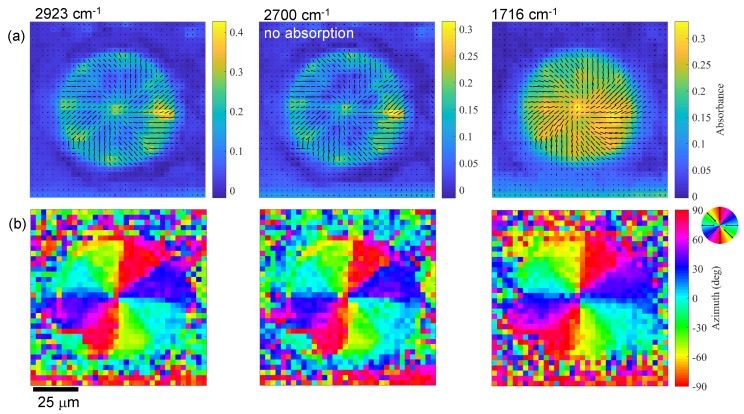
(**a**) Orientation images at different wavenumbers: at the absorption bands and at the wavenumber of 2700 cm^−1^ where there was no absorption peak. The orientation azimuth angle $\theta^{\prime }$ was measured from the fit taken at four θ angles, and the length of the black line represents the orientation strength defined by the amplitude of the min-max absorbance $A_{max}-A_{min}$ [12]. (**b**) Orientation azimuth at the three wavenumbers. Orientation azimuth direction is the same as the polymer structure direction.

Figure 4 shows single wavenumber images at one polarisation (vertical θ = 90°) based on the acquired FPA-FTIR image. With measurements of at least four θ angles, it was possible to recover the orientation of the absorbing dipoles, which follows Malus law $\cos^{2}\theta$ [11]. The resultant orientation maps overlaid with the absorbance are shown in Figure 5a. The azimuth plots (Figure 5b) reveal a well orientated radial pattern, as would be expected for the q=1 plate. Since the same alignment maps were observed for the absorption lines C=O and C–H, as well as for the non-absorbing band at 2700 cm^−1^, the determined alignment was due to the structure of the q-plate rather than the molecular alignment within the polymer. Pixel dimensions (2.8×2.8 µm^2^) are larger than those of the Λ = 2 µm period of the polymerised pattern. The diffraction limit at 2700 cm^−1^ (λ = 3.7 µm) was 0.61λ/NA = 5.6 µm for the used NA=0.4. The data shown in Figure 5 correspond to the case when resolution is lower than the image discretisation provided by the pixel array of the FPA detector.

### 3.3. Brown Silk

Brown silk produced by wild moth (*Antheraea pernyi*) was investigated after a standard degumming procedure, which provided single strands [19]. Chemical images of microtomed longitudinal slices of ∼10 µm thickness embedded in epoxy were acquired using FPA-FTIR microspectroscopy.

**Figure 6:**
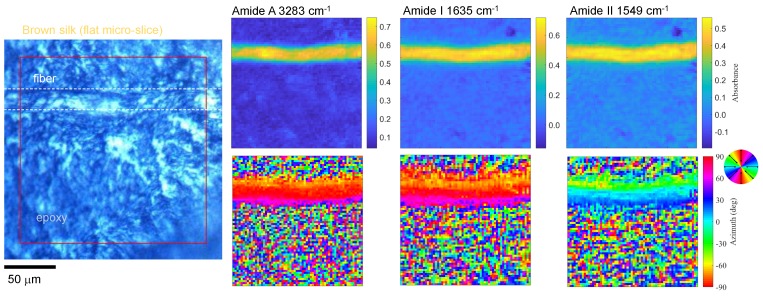
Optical image of a micro-thin brown silk microtome slice and its absorbance and azimuth maps at single wavelengths of specific bands acquired using a thermal IR source; the baselines were integrated from 3700 to 3710 cm^−1^ and from 2000 to 2050 cm^−1^, for the Amide A and Amide I and II bands, respectively.

Figure 6 shows absorbance and azimuth maps for images obtained by the FPA-FTIR technique at four polarisation angles θ at several absorption bands. In the orientation coloured maps, Amide A (N–H) and Amide I (C=O) are mutually aligned along the silk fibre and are perpendicular to the Amide II (C–N) bands. This was an expected result [10], and the flat longitudinal slice was essential for revealing the orientation of the absorbing species. The alignment of the molecular absorbers, in this case, is delivered by a secondary β-sheet structure, which has a periodicity of ∼10 nm, recognisable in the X-ray diffraction (XRD) patterns of silk. The edges of the silk fibre were not well resolved because the diffraction limit of the optics used (NA=0.4) was larger than the pixel size of the FPA; hence, blurring of the silk-epoxy edge occurred. The epoxy region showed low absorbance and random orientation, as expected from an amorphous host.

**Figure 7:**
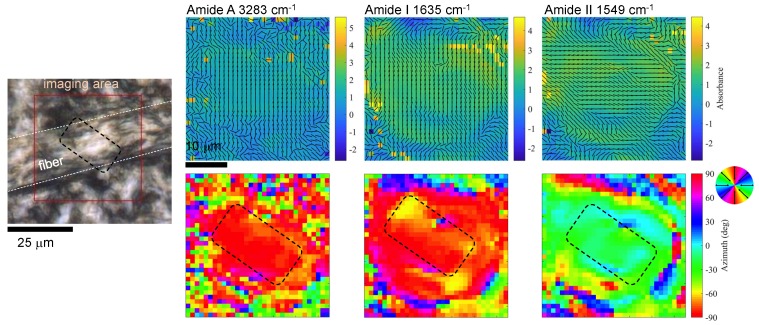
Characterisation of brown silk micro-slice (flat-longitudinal) with synchrotron IR beam. Optical image of a flat longitudinal micro-slice of brown silk fibre in an epoxy matrix and maps of absorbance and azimuth. The black dotted rectangle marks the focal region of focused synchrotron radiation [4].

The same sample was imaged with the FPA-FTIR microspectroscopic technique using the synchrotron IR light source, following the same four θ angle acquisition procedure and calculation of the orientation azimuth. The results (Figure 7) showed a reliable orientation revealed at the focal region. The longer cross section of a rectangular focus [4] had comparable dimensions with the width of the silk fibre slice. The regions out of focus have a high noise. However, the absorbance and orientation can be detected much faster with the FPA detector as compared with a single-point mapping measurement usually employed for scanning over the entire area of interest [20]. Therefore, the demonstrated imaging approach is of particular importance for detection of changes in the sample and can potentially be carried out in real time. A simple estimate of acquisition time savings using 64×64 FPA imaging (parallel) compared with single-pixel mapping (sequential) is 4096 times for a single image. This is a considerable improvement and speeding up of the data collection. Since four angle measurements were required, an improvement by 10^3^ in data collection was gained.

## 4. Discussion

The demonstrated FPA image acquisition in hyperspectral mode is expected to become a powerful research tool. Even without tight focusing (NA=0.4), it was possible to determine the orientation of the transmitted/absorbed light that originated from sub-wavelength features in q=1 plates: a circular metal grating with a period of Λ = 0.2 µm (Figure 2) and polymerised structures with a period of Λ = 2 µm (Figure 5). Natural silk fibres with secondary protein structures such as β-sheets with a structure length of ∼10 nm [21] were revealed using the four angle imaging of the absorbance (Figure 6). This is an important and useful feature of this imaging method when the feature size is not spatially resolved but the orientation of the pattern is determined. The spatial resolution at the wavelength λ is defined by the numerical aperture NA as 0.61λ/NA. However, the polarisation-dependent absorption depends on the orientation of absorbers in the sample and the anisotoropy of absorption can be observed by polariscopy. In the case of isotropic metal structures with nanoscale features, the characteristic dimension $x_{ch}$ (a 100-nm-wide gold grating $x_{ch}=\Lambda /2$; Figure 1) has to be compared with the electron free path length, which is $l_{fp}\approx 38$ nm in gold [22] and is comparable with the optical skin depth for intensity. If the free electron path is larger than the dimensions of the pattern $x_{ch}<l_{fp}$, the quantum effects and the scattering from the pattern edges become important (a particle in the box case). For complex nanoscale patterns and nanoparticles, the light scattering accounting for the amplitude and phase of individual resonators has to be considered for the description polarisation, direction, and intensity. In the case of metallic gratings, the polarisation perpendicular to the mesh beams is transmitted and the one parallel is reflected. The anisotropy of a nanoscale pattern down to the scale of skin depth and $l_{fp}$ should be recognised in the far-field.

**Figure A1:**
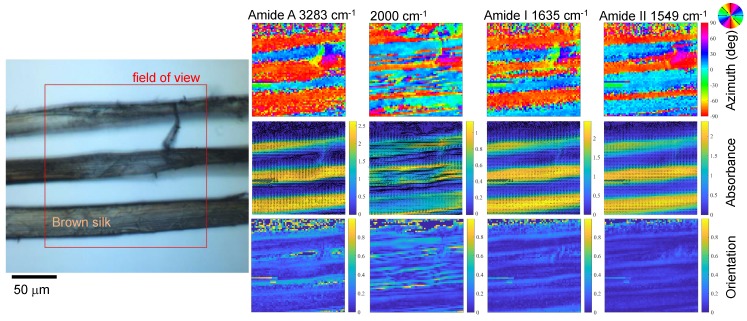
Optical image of brown silk fibres and maps of azimuth, absorbance, and orientation function. Light source was thermal-IR.

**Figure A2:**
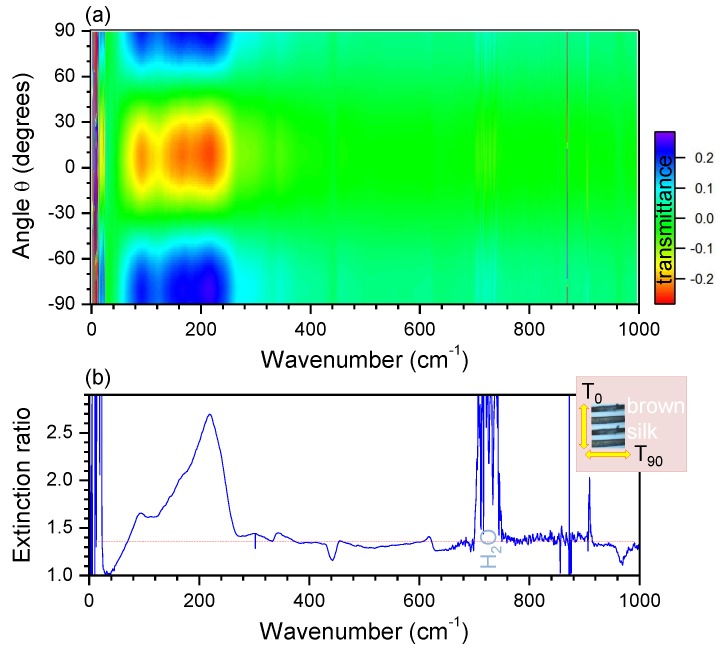
Far-IR measurements of brown silk grating (as shown in the optical image in Figure A1); radiation source was far-IR synchortron beamline. (**a**) The orientational transmittance spectrum map at different polarisation angles θ. (**b**) Extinction ratio (the maximum-to-minimum transmission ratio) for the brown silk grating. The inset shows orientation conventions for the transmittance and extinction ratio $\sigma_{ex}=T_{0}/T_{90}$. Note different wavenumber scales for the panels (**a**,**b**).

The polarisation composition of a synchrotron IR-THz beam is complex, with linear and circular counterparts due to emission occurring at different locations at the edge and inside of a bending magnet [3]. The IR beam is extracted using a mirror with a central slot since synchrotron radiation has a dispersion that is wavelength-dependent. X-rays and UV light are transmitted through the central slot, while visible and IR are reflected off the mirror surface, with longer wavelengths at a larger distance from the centre. Defocusing of IR synchrotron radiation, which is naturally highly focused, into a wider illumination area suitable for use with the FPA detector is a formidable engineering challenge [4]. The spectral and polarisation composition of a focal spot tens-of-µm in cross section is not trivial. This study shows that, even without exact knowledge of polarisation in the beam, it was possible to confirm the orientation of absorbers in silk using measurements with a linear polariser at four θ orientation angles (Figure 6 vs. Figure 7). Non-sliced brown silk fibres were measured with a thermal-IR source at the absorbance bands as well as at 2000 cm^−1^ where no specific absorption peak was present (Figure A1). Due to the circular cross section of silk fibres, the thickness varied at different lateral locations on the image. This reduced the possibility of determining the molecular alignment as compared with the flat micro-slices of silk (Figure 6). It was also revealed that aligned silk fibres were acting as a polariser (Figure A1 and Figure A2).

**Figure A3:**
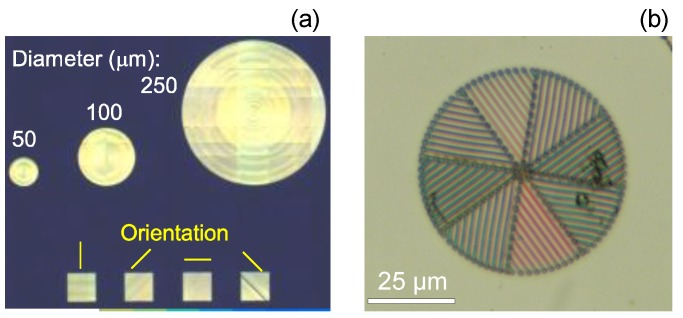
(**a**) Optical microscope image of circular and 1D gratings milled in 100-nm-thick Au on micro-thin (30 µm) sapphire. The smallest circular grating was used in measurement shown in Figure 1. (**b**) A closeup optical image of the q-plate used for measurements shown in Figure 3.

Another insight, which follows from this application of FPA in FTIR hyperspectral imaging is the possibility of carrying out a simultaneous four angle measurement when absorption anisotropy of the sample is known. The nano-gratings milled into a metallic surface (Figure 1) are acting as spectrally anisotropic wire-grid polarisers and can be imaged over the FPA field of view. This provides the possibility of obtaining a four-angle measurement in a single exposure with gratings milled side-by-side at four angles (Figure A3). Certainly, the homogeneity of the sample over the region of measurement should be known for unambiguous data interpretation.

With λ/4-waveplates becoming more available at specific IR wavelengths, a set of four gratings (Figure A3) can be used to determine Stokes parameters [23] $S=\left(I,Q,U,V\right)\equiv \left(S_{0},S_{1},S_{2},S_{3}\right)$ of light, where the total intensity $I={\left|E_{x}\right|}^{2}+{\left|E_{y}\right|}^{2}$ (also a $S_{0}$ parameter), $Q={\left|E_{x}\right|}^{2}-{\left|E_{y}\right|}^{2}$ ($S_{1}$), $U={\left|E_{a}\right|}^{2}-{\left|E_{b}\right|}^{2}$ ($S_{2}$), where a Cartesian basis (x,y) is rotated by 45° to obtain (a,b), and $V={\left|E_{l}\right|}^{2}-{\left|E_{r}\right|}^{2}$ ($S_{3}$), where SAM σ=±1 corresponds to the left (+1) and right (−1) polarisations, respectively. The milled metal sub-wavelength gratings provide a capability for polarisation characterisation, which is still missing over a wide IR-THz spectral range. Polarisation effects are used at shorter visible wavelengths in fluorescence emission spectroscopy [24], where the same milled circular grating (Figure 1) would perform as a sub-wavelength structure.

Figure 1 shows the Malus $\cos^{2}\theta$ pattern along circular grooves, which provides the possibility for determining a local orientation azimuth with a higher (at larger radial locations) and lower (at the centre) fidelity. A single circular grating can perform as four 45° rotated linear gratings at quarter segments and, in a single measurement, can provide the orientation azimuth. By combining the size of the circular grating and optical magnification of the optical setup, in theory, it is possible to realise four-pixel, four-angle detection. This is planned for future work.

## 5. Conclusions and Outlook

FPA-FTIR with the four polarisations method was employed to obtain the orientational dependence of the absorbance in three samples with sub-wavelength features. A circular nano-grating was imaged at wavelengths that are, by one order of magnitude, larger than the diffraction limit, but the orientational information in the image was revealed. The orientation of absorbing dipoles (silk) or that of the structural pattern that is not spatially resolved (q-plates), or the polarisation of the transmitted light (circular nano-grating), was revealed in the azimuthal orientation images obtained at four orientations of the linearly polarised light. The pixel dimensions were smaller than the diffraction limit in all cases. Well-defined radial orientation of the grooves was recovered by the applied four-angle method. We discuss the application of such a grating as a polariser for the four-angle method. In laser-polymerised, azimuthally orientated gratings—the q-plates—the radial pattern was also reliably retrieved in the orientation images. The highest spatial frequency of molecular absorbers was in brown silk, and the orientational azimuth was reliably determined. In addition to the uniform illumination using a thermal-IR source, we also used focused synchrotron-IR irradiation at higher magnification, and the same orientation of the absorption bands in silk was confirmed.

It has been shown that the orientation of dipoles, which are sub-1 nm objects and absorb in the IR spectral window, was determined by polariscopic imaging. The alignment and secondary structure of β-sheets in silk (∼10 nm) and pattern of polymerised rods in q-plates (∼1 µm) was also recovered by polariscopic imaging. The polariscopic method could have implications in surface-enhanced IR absorption spectroscopy (SEIRAS) for the design of patterns, where a higher sensitivity of detection can be achieved in the IR-THz spectral range [25]. Additionally, polarisation properties of perfect absorbers for IR wavelengths [26,27] can be investigated with the proposed method.

## Appendix A. Supplement

Figure A1 shows spectral analysis from degummed brown silk fibres at several spectral locations measured with thermal IR source and FPA. The fibres were not sliced. The orientation function used to evaluate ordering of the absorbing dipoles is directly obtained from:

(A1) $$f_{\psi }=\frac{D-1}{D+2}\times \frac{2}{3\langle \cos^{2}\alpha \rangle -1},$$

where the dichroic ratio $D\equiv A_{max}/A_{min}\equiv A_{\|}/A_{⊥}$ with ‖ orientation corresponding to the α = 0° angle between the molecular chain axis and transition dipole moment, 〈…〉 is the ensemble average; the orientation of the CH_2_ dipole moment is defined here as the molecular chain axis. The range of $f_{\psi }$ values is from 0 to 1 (see orientation panels in Figure A1), where 1 corresponds to the perfect alignment of the molecular chain axis to the orientation direction, and 0 corresponds to the random phase.

The extinction ratio is defined by the maximum-to-minimum transmission ratio $\sigma_{ex}=T_{0}/T_{\pi /2}$ , where $T_{0;\pi /2}$ are the maximum and minimum transmittance values at the two perpendicular orientation angles θ=0,π/2, respectively (Figure A2). The $\sigma_{ex}$ was measured for the silk grating via a wide far-IR spectral range (Figure A2). For the $\sigma_{ex}=1$ material or pattern has isotropic transmittance while the metallic grid polariser has $\sigma_{ex}\gg 1$ since the E-field of light polarised along the metallic lines of the grating (θ = 90°; Figure A2b) is reflected (extinct in transmission) while the transmittance $T_{0}$ is maximal. Since $\sigma_{ex}<1$ was not observed, there was no anisotropy induced by a particular molecular alignment in brown silk grating. The anisotropy of absorbance due to molecular alignment can be quantified from the angular dependence of A(θ) and only four angles with angular separation of π/4 are required [11] to retrieve the orientational dependence $A\left(\theta \right)=A_{0}\cos^{2}\theta +A_{\pi /2}\sin^{2}\theta$. The maximum $\sigma_{ex}\approx 2.5$ was observed at 210±10 cm^−1^ wavenumber. In this range around 48 µm wavelength, the aligned silk fibres of comparable diameter were acting as a polariser with $\sigma_{ex}$ larger as compared with 3D printed polymer gratings which do not have particular molecular ordering [3].

Figure A3a shows an optical image of the circular gratings of different diameters milled into 100-nm-thick Au deposited on 30-µm-thick sapphire. The period of gratings is 200 nm with duty cycle of 0.5. The orientation of gratings milled by Ga^+^ ions is at four orientation angles which can be used as polarisers for four angle method [11] applied in this study or for polariscopy measurements to determine Stokes parameters of an arbitrary polarised light. The panel (b) shows the q-plate polymerised out of SZ2080 resist.

## References

[1] Cheeseman S., Truong V., Vongsvivut J., Tobin M.J., Crawford R., Ivanova E.P. (2018). Applications of Synchrotron-Source IR Spectroscopy for the Investigation of Insect Wings.

[2] Wang D.C., Sun S., Feng Z., Tan W., Qiu C.W. (2018). Multipolar-interference-assisted terahertz waveplates via all-dielectric metamaterials. Appl. Phys. Lett..

[3] Ryu M., Linklater D., Hart W., Balčytis A., Skliutas E., Malinauskas M., Appadoo D., Tan Y., Ivanova E.P., Morikawa J. (2018). 3D Printed Polarising Grids for IR-THz Synchrotron Radiation. J. Opt..

[4] Tobin M., Vongsvivut J., Martin D., Sizeland K., Hackett M., Takechi R., Fimorgnari N., Lam V., Mamo J., Carter E. (2018). Focal plane array IR imaging at the Australian Synchrotron. Infrared Phys. Technol..

[5] Tao H., Kaplan D.L., Omenetto F.G. (2012). Silk Materials: A Road to Sustainable High Technology. Adv. Mater..

[6] Shao Z., Vollrath F. (2002). Surprising strength of silkworm silk. Nature.

[7] Rousseau M.E., Lefevre T., Beaulieu L., Asakura T., Pezolet M. (2004). Study of Protein Conformation and Orientation in Silkworm and Spider Silk Fibers Using Raman Microspectroscopy. Biomacromolecules.

[8] Du N., Liu X.Y., Narayanan J., Li L., Lim M.L.M., Li D. (2006). Design of Superior Spider Silk: From Nanostructure to Mechanical Properties. Biophys. J..

[9] Malinauskas M., Žukauskas A., Bičkauskaitė G., Gadonas R., Juodkazis S. (2010). Mechanisms of three-dimensional structuring of photo-polymers by tightly focussed femtosecond laser pulses. Opt. Express.

[10] Ryu M., Bačytis A., Wang X., Vongsvivut J., Hikima Y., Li J., Tobin M.J., Juodkazis S., Morikawa J. (2017). Orientational Mapping Augmented Sub-Wavelength Hyper-Spectral Imaging of Silk. Sci. Rep..

[11] Hikima Y., Morikawa J., Hashimoto T. (2011). FT-IR Image Processing Algorithms for In-Plane Orientation Function and Azimuth Angle of Uniaxially Drawn Polyethylene Composite Film. Macromolecules.

[12] Honda R., Ryu M., Balčytis A., Vongsvivut J., Tobin M.J., Juodkazis S., Morikawa J. (2019). Paracetamol micro-structure analysis by optical mapping. Appl. Surf. Sci..

[13] Wang X., Kuchmizhak A.A., Brasselet E., Juodkazis S. (2017). Dielectric geometric phase optical elements fabricated by femtosecond direct laser writing in photoresists. Appl. Phys. Lett..

[14] Marrucci L., Karimi E., Slussarenko S., Piccirillo B., Santamato E., Nagali E., Sciarrino F. (2011). Spin-to-orbital conversion of the angular momentum of light and its classical and quantum applications. J. Opt..

[15] Slussarenko S., Murauski A., Du T., Chigrinov V., Marrucci L., Santamato E. (2011). Tunable liquid crystal q-plates with arbitrary topological charge. Opt. Express.

[16] Biener G., Niv A., Kleiner V., Hasman E. (2002). Formation of helical beams by use of Pancharatnam - Berry phase optical elements. Opt. Lett..

[17] Hakobyan D., Brasselet E. (2014). Left-handed optical radiation torque. Nat. Photonics.

[18] Rekštytė S., Jonavicius T., Gailevičius D., Malinauskas M., Mizeikis V., Gamaly E.G., Juodkazis S. (2016). Nanoscale precision of 3D polymerisation via polarisation control. Adv. Opt. Mat..

[19] Balčytis A., Ryu M., Wang X., Novelli F., Seniutinas G., Du S., Wang X., Li J., Davis J., Appadoo D., Morikawa J., Juodkazis S. (2017). Silk: Optical Properties over 12.6 Octaves THz-IR-Visible-UV Range. Materials.

[20] Ryu M., Kobayashi H., Balčytis A., Wang X., Vongsvivut J., Li J., Urayama N., Mizeikis V., Tobin M., Juodkazis S. (2017). Nanoscale chemical mapping of laser-solubilized silk. Mat. Res. Express.

[21] Ryu M., Honda R., Cernescu A., Vailionis A., Balcytis A., Vongsvivut J., Li J.L., Linklater D.P., Ivanova E.P., Mizeikis V., Tobin M.J., Morikawa J., Juodkazis S. (2019). Nanoscale optical and structural characterisation of silk. arXiv.

[22] Gall D. (2016). Electron mean free path in elemental metals. J. Appl. Phys..

[23] McMaster W.H. (1954). Polarization and the Stokes Parameter. Am. J. Phys..

[24] Stedmon C.A., Bro R. (2008). Characterizing dissolved organic matter fluorescence with parallel factor analysis: A tutorial. Limnol. Oceanogr. Methods.

[25] Zhizhchenko A., Kuchmizhak A., Vitrik O., Kulchin Y., Juodkazis S. (2018). On-demand concentration of an analyte on laser-printed polytetrafluoroethylene. Nanoscale.

[26] Xiong X., Jiang S., Hu Y., Peng R., Wang M. (2013). Structured Metal Film as a Perfect Absorber. Adv. Mat..

[27] Nishijima Y., Balčytis A., Naganuma S., Seniutinas G., Juodkazis S. (2018). Tailoring Metal and Insulator Contributions in Plasmonic Perfect Absorber Metasurfaces. ACS Appl. Nanomater..

